# Statins as Adjuvant Therapy for COVID-19 to Calm the Stormy Immunothrombosis and Beyond

**DOI:** 10.3389/fphar.2020.579548

**Published:** 2021-01-19

**Authors:** Alpo Vuorio, Petri T. Kovanen

**Affiliations:** ^1^ Mehiläinen Airport Health Centre, Vantaa, Finland; ^2^ Department of Forensic Medicine, University of Helsinki, Helsinki, Finland; ^3^ Wihuri Research Institute, Helsinki, Finland

**Keywords:** atherosclerosis, cardiovascular disease, coronavirus, COVID.19, immune response, statins

## Introduction

Cytokine storm is a severe immune response that can be triggered by the coronavirus SARS-CoV-2 infection in susceptible patients, and, in the severe form of the COVID-19 disease, it is potentially lethal because of its systemic immunothrombogenic sequelae (Cron, 2020). The cytokine storm consists of excessive macrophage activation, haemophagocytic lymphohistiocytosis, and a syndrome characterized by excessive release of proinflammatory cytokines (Henderson et al., 2020). Clinical trials are underway to investigate, besides anti-viral agents, the use of appropriate immunosuppressive and immunomodulatory drugs, and also specific drugs to target individual pro-inflammatory cytokines (Soy et al., 2020). Since we still lack efficient means for managing the cytokine storm, there is a need for drugs that can potentially mitigate some of the downstream effects of the potentially deadly immune response. Toward this end, the widely used statins may be considered as adjuvant drugs in the treatment of severe COVID-19. The statins may ameliorate, at least partially, some components of the cytokine storm and its sequelae, which are related to poor prognosis in COVID-19 patients.

## Predictors of In-Hospital Mortality in COVID-19 Patients

The strongest predictors of in-hospital mortality in COVID-19 patients include elevated interleukin -6 (IL-6) and D-dimer levels at hospital admission (Cummings et al., 2020; Nadkarni et al., 2020). Indeed, a recent systematic review and meta-analysis showed that serum D-dimer concentrations in patients with severe COVID-19 are significantly higher when compared to those with non-severe forms (Paliogiannis et al., 2020). Also, increased levels of fibrinogen (Terpos et al., 2020) and IL-1 (Shakoory et al., 2016), and an elevated neutrophil-to-lymphocyte ratio (NLR) have been associated with poor prognosis in COVID-19 patients (Ciccullo et al., 2020).

The level of IL-6 has previously been used as a biomarker of viral virulence (Velazquez-Salinas et al., 2019). IL-6 possesses marked proinflammatory properties (Moore and June, 2020) and it is possible that IL-6 blockade, for example with the immunosuppressive drug tocilizumab (Rose-John et al., 2017; Quartuccio et al., 2020), may have the potential to reduce viral virulence. In a very recent study, it was found that in hospitalized patients with COVID-19 tocilizumab treatment was associated with fewer serious infections; yet, it was not effective for preventing intubation or death in the moderately ill COVID-19 patients studied (Stone et al., 2020).

High levels of circulating D-dimers have been associated with an elevated risk of thrombosis (Leonard-Lorant et al., 2020; Mucha et al., 2020). It is important to note that some COVID-19 patients have underlying diseases that may increase the risk for bleeding, and thus caution should be exercised during anticoagulation (Wang et al., 2020). In COVID-19 patients, the pathophysiology of thrombosis is largely driven by the infectious immunoinflammatory process that occurs systemically in veins, arteries, and the microvasculature of vital organs, such as the lungs, kidneys, heart, and brain (Siddiqi et al., 2020). In the vascular system, the endothelium is the primary target of the immune-inflammatory attack, and, when attacked, the endothelial cells tend to lose their antithrombotic properties (Belen-Apak and Sarıalioğlu, 2020; Libby and Lüscher 2020). The endothelial cells can also be infected with the coronavirus. Such infectious endothelial damage, termed endotheliitis, has been observed both in the myocardial and cerebral vessels, and due to the ensuing local thrombus formation has led to severe ischemic syndromes (Crippa et al., 2020; Mosleh et al., 2020; Varga et al., 2020).

## D-Dimers, Fibrinogen, and the Endothelium

D-dimers are fibrin degradation fragments that are generated upon fibrinolysis of a blood clot, i.e., their presence in the blood reflects coagulation activation with ensuing formation of blood clots and the accompanying clot degradation by fibrinolysis. Then, understandably, the magnitude of an increased D-dimer level discloses the extent of thrombus formation and also predicts the clinical severity of any thrombotic complication associated with a disease, such as deep vein thrombosis (Andreescu et al., 2002). In a study of patients with suspected pulmonary embolism (156 statin users and 147 antiplatelet drug users), statin use was associated with a modest 15% decrease in D-dimer levels (95% confidence interval [CI] −28 to −0.6%) whereas the use an antiplatelet drug (mainly acetylsalicylic acid and clopidogrel) had no significant effect (Schol-Gelok et al., 2018). Similarly, in a cohort study including 6,814 male and female subjects without cardiovascular disease (aged 45 to 84 years), the D-dimer levels were found to be 9% lower in statin users than in non-users (Adams et al., 2013).

Statin use does not seem to affect fibrinogen levels (Dujovne, et al., 2000; Sbarouni et al., 2000). However, treatment with statins can lead to a significant downregulation of the blood coagulation cascade as a result of decreased tissue factor expression, which, again, leads to reduced thrombin generation and attenuation of procoagulant reactions catalyzed by thrombin, such as fibrinogen cleavage (Undas et al., 2005; Undas et al., 2014). Since in the patients with COVID-19, the endothelial cells are the target of the cytokine storm and may also become infected by the virus, the dysfunctional endothelial cells lose their antithrombotic surface properties (Libby and Lüscher 2020). Indeed, the endothelial dysfunction with ensuing organ hypoxia may be the hardest challenge regarding the cardiovascular consequences in COVID-19 patients. On the other hand, the ability of statins to improve endothelial function (Masoura et al., 2011) could at least partially ameliorate the prothrombotic state of the endothelium.

## Interleukin-6 and Interleukin-1

The anti-inflammatory effects of statins have been studied using a cytokine-mediated interaction model of human vascular smooth muscle cells and mononuclear cells in culture (Loppnow et al., 2011). In this cell culture study, simvastatin, atorvastatin, fluvastatin, and pravastatin reduced IL-6 production by 53, 50, 64, and 60%, respectively. This finding suggests that, if translatable for *in vivo* applications, statins may be able to reduce the pro-inflammatory effects of IL-6 in tissues. However, no clinically significant reduction in the concentration of circulating IL-6 has been observed among statin users (Wiklund et al., 2002; Lyngdoh et al., 2011). Concerning IL-1, simvastatin use is associated with a decreased concentration of IL-1β in gingival crevicular fluid in patients with inflammatory periodontal disease (Cicek et al., 2016). The promising role of statins as inhibitors of IL-1β synthesis and release warrants further investigation (Liberale et al., 2019). Among the strategies to inhibit the effects of cytokines, blocking the IL-1 receptor has been particularly beneficial, as shown in a controlled study in sepsis patients with the macrophage activation syndrome (Shakoory et al., 2016).

## Neutrophil—Lymphocyte Ratio

In the Danish General Suburban Population Study, inflammatory markers were analyzed in 2,922 statin users and 16,873 non-users (Sørensen et al., 2019). In this study, the neutrophil-lymphocyte ratio was reduced by 3% among statin users (95% CI 1 to 5%, *p* = 0.003). In an earlier study, initiation of statin treatment did not affect the neutrophil-lymphocyte ratio in hypercholesterolemic patients (Gungoren et al., 2016). Accordingly, we can state that, based on the available data, statins appear to have no or only a modest effect on the neutrophil-lymphocyte ratio.

## Other VIRAL Infections

In a study of 3,043 hospitalized laboratory-confirmed influenza patients of whom one-third received statin treatment, the authors assessed the effect of statin administration before or during hospitalization using a multivariable logistic regression model (Vandermeer et al., 2012). In the cited study, an adjustment was made for age and race, as well as for cardiovascular, pulmonary, and renal disease, and influenza vaccination, and the authors found that statin consumption before or during hospitalization decreased the risk of death (adjusted odds ratio 0.59; 95% CI 0.38 to 0.92). A beneficial effect of statins has also been suggested for patients with Middle Eastern respiratory syndrome (MERS), a viral illness also caused by a coronavirus (Yuan, 2015).

## COVID-19 and Statins

Analysis of in-hospital deaths among 8910 COVID-19 patients from Asia, Europe, and North America revealed that statin use was associated with a favorable prognosis (Mehra et al., 2020). In a recent retrospective study among 154 COVID-19 patients in nursing homes in Belgium, De Spiegeleer et al. found a significant positive association between statin use and the absence of symptoms (OR 2.91; CI 1.27 to 6.71, *p* = 0.011), and the result remained significant after adjustment for age, sex, functional status, diabetes mellitus, and hypertension (De Spiegeleer et al., 2020). However, in this study, the effects of statin use on serious clinical outcomes did not reach statistical significance. The authors concluded that statins may be associated with a beneficial effect on COVID-19-related symptoms in old and frail persons and suggested that a potentially favorable interaction between statins and the drugs regulating the renin-angiotensin system should be further investigated. Such interaction may emerge, as in COVID-19 patients the use of either an angiotensin-converting enzyme inhibitor or a statin was associated with a lower risk of in-hospital death when compared with COVID-19 patients who did not use either class of drugs (Fedson et al., 2020; Mehra et al., 2020). To note, statin therapy has been demonstrated to associate with significant improvement in both peripheral and coronary endothelial function (Reriani et al., 2011). This seminal clinical observation helps us to understand the benefit of statin use under conditions of endothelial stress, such as occurs during infection.

Two meta-analyses on the association between statin use and COVID-19 have been published recently. In the smaller meta-analysis, association between statin use and in-hospital outcomes of COVID-19 was analyzed until August 1, 2020 by systematically searching the Google Scholar database (Hariyanto and Karniawan 2020). A total of nine studies with a total of 3,449 patients were included in the analysis. This meta-analysis showed that statin use did not improve the severity outcome (OR = 1.64; 95% CI 0.51–5.23) or the mortality rate from COVID-19 (OR = 0.78; 95% CI 0.50–1.21). Thus, no statin-dependent benefit could be demonstrated. Also, the larger meta-analysis has been carried out among hospitalized COVID-19 patients (Kow & Hasan, 2020). In this comprehensive meta-analysis, the risk of severe illness and/or mortality in COVID-19 among statin users was compared to non-statin users (total number of patients 8,990). The authors searched PubMed, Google Scholar, and medRxiv (preprint repository) databases up to July 27, 2020, and the pooled analysis revealed that among the COVID-19 patients with statin treatment, not only the severity of the illness but also the mortality was significantly reduced (HR = 0.70; 95% CI 0.53–0.94).

## Discussion

Increasing evidence supports the use of statins in patients with COVID-19 (Bifulco and Gazzerro, 2020; Castiglione et al., 2020; Dashti-Khavidaki and Kahlili, 2020). Accordingly, the National Institutes of Health COVID-19 Treatment Guidelines recommend that patients with COVID-19 who are prescribed statins for the treatment or prevention of cardiovascular disease should continue statin therapy (National Institutes of Health, 2020). Statins are generally safe and are cost-effective; yet, this class of drugs is underused (Chen et al., 2019). Importantly, a recent retrospective analysis of SARS-CoV-2 infection related mortality in hospitalized patients with COVID-19 not only confirmed the beneficial background of statin therapy but also revealed that maintenance of statin therapy during hospitalization correlated with an even better prognosis (Masana et al., 2020). Based on the currently available data, we consider that a patient with diagnosed COVID-19 should continue statin use as prescribed; in addition, the short- and long-term adherence to and persistence with statin therapy should be improved in patients with a low level of adherence. Moreover, if a statin-naïve adult patient with cardiovascular disease risk factors fulfills the criteria for statin therapy, the diagnosis of COVID-19 should act as an additional trigger for immediate initiation of statin therapy. Even, if the statin-naïve COVID-19 patient who may not fulfill all the criteria for statin treatment according to present guidelines (Cholesterol Treatment Trialists’ (CTT) Collaborators, 2012), initiation of therapy should nevertheless be considered, as the endothelial-damaging action of the viral infection increases the risk of thrombotic complications, while statins tend to improve the function of the endothelium under stress (Reriani et al., 2011). Suitable candidates for statin therapy are middle-aged COVID-19 patients in particular, since many of them may have subclinical coronary atherosclerosis at LDL-cholesterol levels currently considered normal and even in the absence of other cardiovascular disease risk factors (Fernández-Friera et al., 2017; López-Melgar et al., 2020).

Another important reason for initiating permanent statin treatment is that the development of atherosclerotic lesions is accelerated during infection and inflammation (Mehta et al., 1998), and it is likely that COVID-19 triggers a sustained increase in the risk of cardiovascular disease, at least in patients with genetically elevated plasma cholesterol levels (Vuorio et al., 2020). It was recently estimated that approximately 5% of COVID-19 patients will experience an acute ischaemic stroke, and those with multiple-organ dysfunctions are at even higher risk of an acute stroke (Qureshi et al., 2020). It is noteworthy that the safety of statins has been shown in children with familial hypercholesterolemia aged 8 years and above (Vuorio et al., 2019). In children aged 3 to 17 years with H1N1 influenza virus infection and severe clinical manifestations of the infection, IL-1β and IL-6 plasma levels were significantly upregulated when compared to children with H1N1 and mild symptoms (Chiaretti et al., 2013).

Numerous mechanisms have been proposed to underlie the favorable effects of statins in COVID-19. One such mechanism is their mild anticoagulant effect with a potential to decrease the risk of thrombus formation in the veins, arteries, and microvessels (Undas et al., 2014), which individually or jointly are considered the primary causes of the frequently fatal respiratory and cardiovascular failures in COVID-19 patients. COVID-19 may trigger a sustained accelerated progression of atherosclerosis during the recovery phase and beyond, emphasizing the importance of the continual use of statins (Vuorio et al., 2020). There is an urgent need to collect data related to the cardiometabolic and the immunothrombotic status of hospitalized patients with COVID-19 who have received or have not received statin therapy. Analysis of such information will enable us to test the hypothesis that the statin drugs alleviate the macrovascular cardiovascular disease and its atherothrombotic complications (acute myocardial infarction and ischemic stroke) in COVID-19 patients. Such data are necessary also for the critical evaluation of the suggested beneficial effects of statins on the immunothrombotic component of COVID-19 caused by the systemic endothelial dysfunction in the entire circulatory system in patients with the illness (Vuorio and Kovanen, 2020). Such a study of the potential beneficial effects of statin treatment before, during, and after the development of the cytokine storm should include COVID-19 patients with and without traditional cardiovascular disease risk factors, notable hypercholesterolemia. Only then will it be possible for us to learn whether the beneficial effects of statins on multiple molecular targets on their pleiotropic and/or their plasma cholesterol-lowering properties. Most importantly, however, such therapeutic strategies should disclose the real value of statins as adjuvant therapy in the prevention and treatment of the stormy immunothrombosis in COVID-19 patients.

## Author Contributions

AV: writing the first draft; AV and PK: reviewing and editing to produce the final draft (equal contribution).

## Conflict of Interest

PK has received consultancy fees, lecture honoraria and/or travel fees from Amgen, Novartis, Raisio Group, and Sanofi.

The remaining author declares that the research was conducted in the absence of any commercial or financial relationships that could be construed as a potential conflict of interest.
